# The Efficacy of Albumin Infusion in Septic Patients with Hypoalbuminemia: An International Retrospective Observational Study

**DOI:** 10.3390/jcm14134790

**Published:** 2025-07-07

**Authors:** Hsin-Yu Liu, Yu-Ching Chen, Ju-Fang Liu, Pei-Sung Hsu, Wen-Pin Cheng, Shih-Sen Lin

**Affiliations:** 1 Division of General Medicine, Department of Medical Education, Shin Kong Wu Ho-Su Memorial Hospital, Taipei City 111045, Taiwan; jill10230433@gmail.com; 2 Independent Researcher, Taipei City 106409, Taiwan; lexiechen0919@gmail.com; 3 School of Oral Hygiene, College of Oral Medicine, Taipei Medical University, Taipei City 110301, Taiwan; jufangliu@tmu.edu.tw; 4 Division of Chest Medicine, Department of Internal Medicine, Shin Kong Wu Ho-Su Memorial Hospital, Taipei City 111045, Taiwan; pack14tw@gmail.com; 5 Department of Research, Shin Kong Wu Ho-Su Memorial Hospital, Taipei City 111045, Taiwan; t011788@ms.skh.org.tw; 6 Graduate Institute of Human Resource and Knowledge Management, National Kaohsiung Normal University, Kaohsiung City 802561, Taiwan; 7 Clinical Trial Center, Department of Research, Shin Kong Wu Ho-Su Memorial Hospital, Taipei City 111045, Taiwan; 8 Graduate Institute of Clinical Medicine, College of Medicine, National Taiwan University, Taipei City 106319, Taiwan

**Keywords:** sepsis, hypoalbuminemia, albumin supplementation

## Abstract

**Background/Objectives**: Albumin supplementation is widely used for hypoalbuminemia treatment in patients with critical illness, especially those with cirrhosis. However, studies have demonstrated that routine albumin administration is not always advantageous. We examined how albumin supplementation affects survival outcomes in patients with sepsis with hypoalbuminemia. **Methods**: This study was conducted by researchers in Taiwan using data from the TriNetX research platform, covering the period from 1 April 2014 to 30 April 2024. This platform aggregates real-world data from healthcare organizations worldwide. From this dataset, 1,147,433 patients who developed sepsis and hypoalbuminemia with albumin levels <3.5 g/dL were identified. The study population was stratified into two groups on the basis of whether they received albumin infusion or not. To compare outcomes, hazard ratios (HRs) and 95% confidence intervals (CIs) were calculated between propensity-score-matched patients who did and did not receive albumin supplementation. Subgroup analysis by albumin levels was conducted. **Results**: Albumin infusion was linked to increased risks of 30-day mortality (HR [95% CI] = 1.800 [1.774–1.827], *p* < 0.05), shock (HR [95% CI] = 1.436 [1.409–1.465], *p* < 0.05), septic shock (HR [95% CI] = 1.384 [1.355–1.415], *p* < 0.05), hypovolemic shock (HR [95% CI] = 1.496 [1.391–1.608], *p* < 0.05), cardiogenic shock (HR [95% CI] = 1.553 [1.473–1.637], *p* < 0.05), heart failure (HR [95% CI] = 1.098 [1.080–1.116], *p* < 0.05), and pulmonary edema (HR [95% CI] = 1.479 [1.438–1.520], *p* < 0.05). The subgroup analysis by albumin levels revealed a trend of increased mortality risk with albumin supplementation in patients with high baseline albumin levels. **Conclusions**: Patients with sepsis with hypoalbuminemia who received albumin supplementation exhibited high 30-day mortality rates and increased risks of shock, heart failure, and pulmonary edema compared with those who did not. These findings indicate that routine albumin administration may be linked with unfavorable outcomes in these patients.

## 1. Introduction

Hypoalbuminemia is a significant predictor of both mortality and morbidity in critically ill patients because of its association with circulatory collapse resulting from decreased oncotic pressure and edema. It is commonly observed among hospitalized patients. One study reported that over 70% of hospitalized elderly individuals were affected [1]. The causes of hypoalbuminemia include inflammatory conditions, nutritional deficiencies, renal losses, gastrointestinal losses, burns, cardiac failure, late pregnancy, lactation, and others [2,3]. Management focuses on addressing the underlying cause, as it is typically a secondary manifestation of another disease. Additionally, hypoalbuminemia serves as a prognostic marker for disease severity and mortality risk in hospitalized patients [3]. Hence, albumin supplementation is widely employed in various clinical scenarios. However, albumin infusion for the treatment of hypoalbuminemia was reported to exert adverse effects in certain patient populations [4]. Currently, albumin infusion is recommended in specific clinical situations, such as spontaneous bacterial peritonitis, hepatorenal syndrome, and large-volume paracentesis in liver cirrhosis [5]. Nevertheless, albumin supplementation had no benefits in mortality to certain patient populations, including patients undergoing severe head injury and patients with burns [6,7,8]. In patients with severe traumatic brain injury, administering albumin for resuscitation is linked to a rise in intracranial pressure within the first week [9]. In burn patients, hypoalbuminemia arises from reduced liver synthesis following resuscitation and primarily reflects the severity of illness. Although intravenous albumin may normalize serum levels, it has limited effect on altering the underlying disease course [8]. Because albumin supplementation is associated with a high cost, routine administration is generally not recommended. Given the aforementioned findings, the use of albumin infusion for the treatment of hypoalbuminemia is a topic of ongoing debate.

Considering the robust link between decreased albumin levels and inflammation [10] and given that sepsis can induce hypoalbuminemia and worsen its severity through various pathophysiological mechanisms, the current study focused on examining the efficacy of albumin supplementation in managing hypoalbuminemia in patients with sepsis [11].

Although numerous randomized controlled trials have explored the role of albumin administration in patients with sepsis or septic shock, its clinical benefits remain uncertain. The Saline versus Albumin Fluid Evaluation (SAFE) study analyzed 1218 patients with severe sepsis from an initial dataset. They reported that albumin therapy was linked to a lower odds ratio of death. However, no significant differences were found in secondary outcomes, such as length of stay in the intensive care unit (ICU) and hospital, duration of mechanical ventilation, and incidence of renal replacement therapy, between the groups [4]. The ALBIOS trial showed that adding albumin to crystalloid therapy did not improve 28-day or 90-day survival outcomes compared to using crystalloids alone. Although albumin administration led to minor improvements in hemodynamic parameters, including a slight reduction in heart rate and a marginal increase in mean arterial pressure, these changes were not considered clinically meaningful [12]. A meta-analysis has suggested that albumin may reduce 90-day mortality in patients with septic shock, and potentially improve survival in those with sepsis compared to crystalloids [13]. A study indicated that albumin may be the preferred colloid for resuscitating patients with sepsis and septic shock, although no definitive guidance is available for transitioning from crystalloid to colloid use during resuscitation [14]. In consideration of the aforementioned observations, we investigated whether albumin supplementation is advantageous in patients with sepsis with hypoalbuminemia.

For the uncertainty of the clinical benefit of albumin in septic patients with hypoalbuminemia, we sought to clarify whether albumin infusion is associated with improved outcomes. We hypothesized that albumin infusion may be associated with clinically unfavorable outcomes to patients with sepsis with hypoalbuminemia. We mainly examined overall mortality and the risks of shock, septic shock, cardiogenic shock, hypovolemic shock, heart failure, and pulmonary edema in patients with sepsis with hypoalbuminemia following albumin supplementation.

## 2. Materials and Methods

This retrospective observational study was conducted by researchers in Taiwan using data from the TriNetX research platform, which is a federated cloud-based network that provides access to de-identified electronic health record data from a diverse range of healthcare organizations. The dataset contains a wide range of medical data, including demographics, diagnoses (coded as per International Classification of Diseases, Tenth Revision, Clinical Modification [ICD-10-CM]), procedures (coded either in ICD-10-PCS or Current Procedural Terminology), medications (categorized using the Veterans Affairs National Formulary), laboratory tests (coded in Logical Observation Identifiers Names and Codes), genomics (annotated using the nomenclature of the Human Genome Variation Society), and healthcare utilization patterns, enabling real-time cohort selection and comparative analyses. All information was derived directly from the encounter-level data available within the TriNetX platform. These data are structured and time-stamped, allowing us to assess utilization trends without the need for indirect inference or code-based approximations [15].

Specifically, this study used the dataset from the Global Collaborative Network, which aggregated medical records from multiple healthcare organizations worldwide. Data for the period from 1 April 2014 to 30 April 2024 were included in the analysis. The patient cohort was first accessed and collected using a code query on 8 November 2024. The study included patients aged 18 years or older. The covariates for both the propensity score matching (PSM) and Cox models are listed in Table 1. They were selected to balance sepsis severity and factors contributing to hypoalbuminemia, such as laboratory indicators in Sequential Organ Failure Assessment (SOFA) score, renal albumin loss including nephrotic syndrome, gastrointestinal loss, and burns. Patients with burns and traumatic brain injury were excluded by skin disease and central nervous system disease based on prior evidence suggesting potential harm from albumin in these conditions. Given the large sample size, multiple systemic comorbidities were also included to reduce confounding from underlying disease. The research protocol was approved by the Institutional Review Board of Shin Kong Wu Ho-Su Memorial Hospital (IRB No: 20250307R).

**Figure 1:**
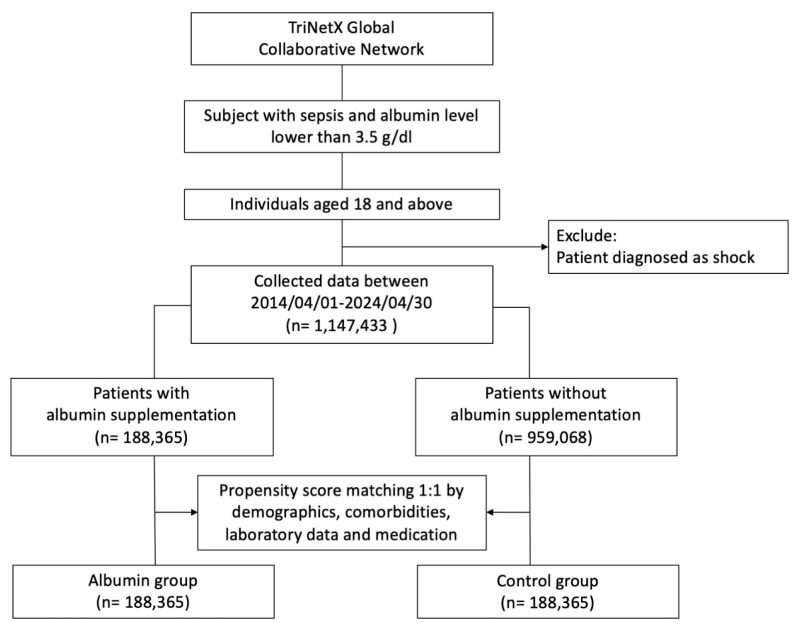
Flowchart of the cohort construction.

Figure 1 provides the flowchart of the establishment of the cohort from the 1,147,433 patients enrolled in the database between 1 April 2014 and 30 April 2024. The study cohort included patients with diagnoses of sepsis and hypoalbuminemia. Patients were included who had sepsis and albumin levels of 0–3.5 g/dL. Patients with any cause of shock were excluded. We stratified the included patients into two groups: an albumin infusion group, which received albumin infusion for the treatment of sepsis and hypoalbuminemia, and a control group, which did not receive albumin infusion. The albumin infusion and control groups comprised 188,365 and 959,068 patients, respectively. The primary outcome was 30-day mortality, defined as the number of deaths occurring within 30 days following albumin administration. The secondary outcomes were the incidence of shock, septic shock, cardiogenic shock, hypovolemic shock, heart failure, and pulmonary edema. All outcomes were defined based on ICD-10 diagnostic codes.

Propensity score matching was employed to balance the baseline characteristics between the two groups and to reduce the effects of confounders. The two groups were matched at a 1:1 ratio without replacement by using a logistic regression model, with treatment assignment used as the dependent variable. After PSM, a total of 188,365 patients who received albumin infusion and 188,365 controls (who did not receive albumin infusion) were selected for inclusion. Individuals from the cohort were longitudinally followed up for 30 days to estimate the risk of mortality (Figure 1).

Statistical analysis of the primary and secondary outcomes was conducted by using the integrated analytical functionalities of the TriNetX platform, which provides built-in support for propensity score matching (PSM), Kaplan–Meier survival analysis, and Cox proportional hazards models.

Survival analyses were performed using time-to-event methodologies, namely Kaplan–Meier curves and Cox proportional hazard models, which were applied for calculating hazard ratios (HRs) with their corresponding confidence intervals. A *p* value of 0.05 was considered to indicate significance. A caliper width of 0.1 standard deviations of the logit of the propensity score was used to ensure close matches between treated and control subjects. Matching quality was assessed using standardized mean differences (SMDs), with an SMD less than 0.1 considered indicative of an adequate balance between groups.

In subgroup analyses, we investigated the effects of albumin supplementation on patients with varying degrees of hypoalbuminemia, with 1.0, 2.0, 3.0, and 3.5 g/dL used as cutoff values.

To strengthen the reliability of our findings, we conducted sensitivity analyses using both unadjusted and fully adjusted Cox proportional hazards models following propensity score matching. The fully adjusted model included all relevant covariates, such as demographics, comorbidities, laboratory values, and pharmacological treatments.

All search criteria (ICD-10 codes, RxNorm medications, time windows, and exclusion rules), the complete analytic workflow, and the aggregated, de-identified output generated by TriNetX are provided in Supplementary Material S1.

## 3. Results

### 3.1. Patient Population and Outcomes

**Figure 2:**
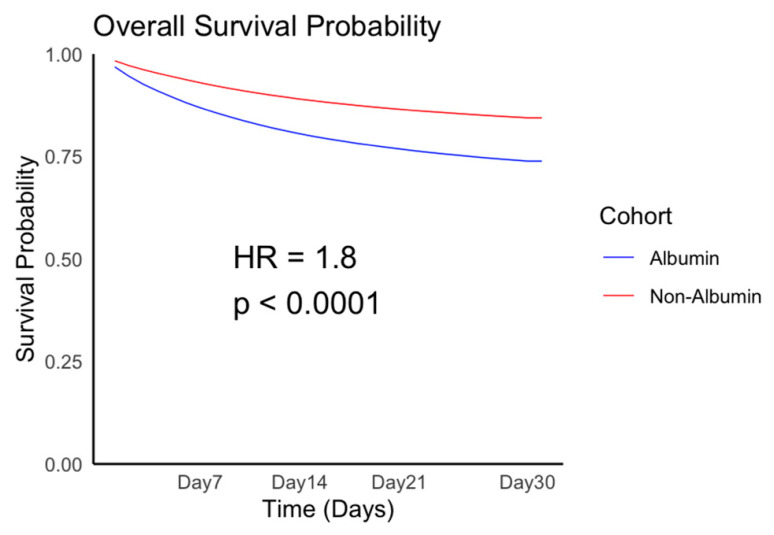
The Kaplan–Meier curve of the survival probability of mortality.

Table 1 presents the demographics of the albumin infusion and control groups both before and after PSM. After PSM, the mean age in the albumin group was approximately 63.6 years, and 43.4% of the included patients were women. The majority of the study population comprised White individuals (60%). The differences in baseline demographics, comorbidities, laboratory data, and medication use between the two groups were minimal, and the two groups were well-matched. We estimated the risks of mortality, shock, heart failure, and pulmonary edema in the albumin infusion and control groups. Regarding the primary outcome, a higher risk of 30-day mortality was noted in the albumin infusion group than that in the control group. Figure 2 presents the Kaplan–Meier curve of the survival probability for patients with sepsis with hypoalbuminemia (Figure 2).

#### Secondary Outcome

**Figure 3:**
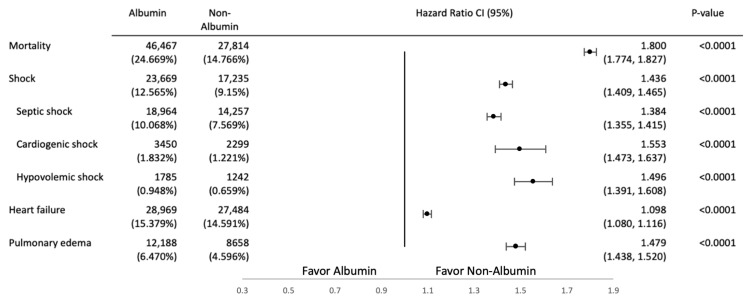
A comparison of primary outcomes between the albumin intervention group and the control group.

The HRs for septic shock, cardiogenic shock, hypovolemic shock, heart failure, and pulmonary edema were higher in the albumin infusion group than in the control group. Figure 3 provides the results of a comparison of the primary outcome (Figure 3).

### 3.2. Subgroup Analysis

**Figure 4:**
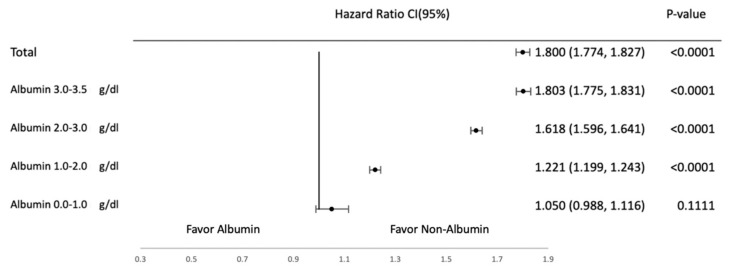
Mortality in subgroup analysis based on albumin levels.

**Figure 5:**
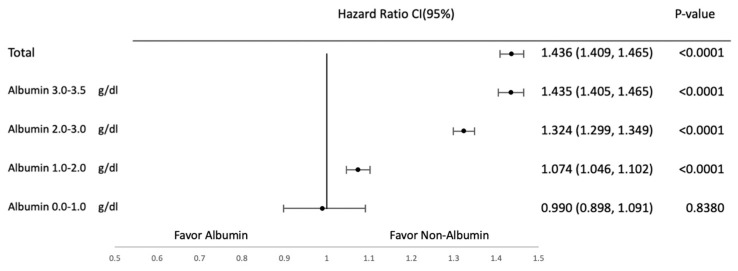
Shock in subgroup analysis based on albumin levels.

**Figure 6:**
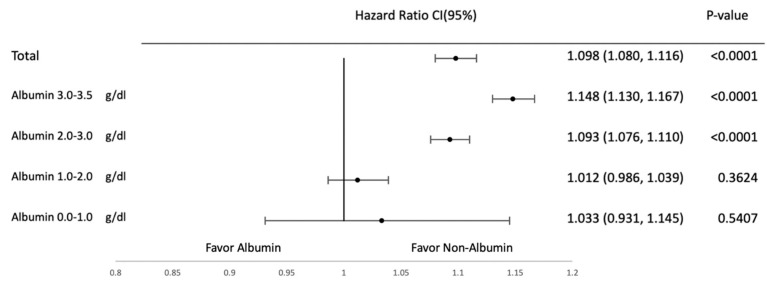
Heart failure in subgroup analysis based on albumin levels.

**Figure 7:**
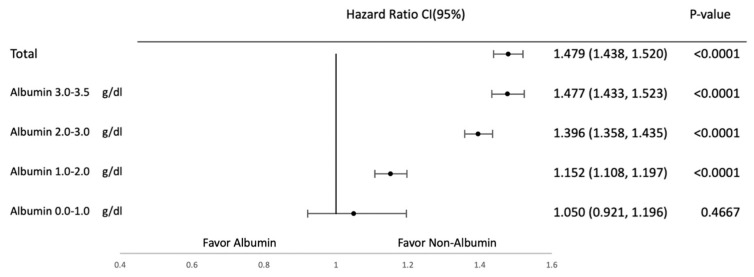
Pulmonary edema in subgroup analysis based on albumin levels.

Subgroup analyses were conducted to evaluate the effects of albumin supplementation across different albumin levels at baseline. They were categorized into the following subgroups on the basis of albumin levels: 0–1, 1–2, 2–3, and 3–3.5 g/dL. Figure 4, Figure 5, Figure 6 and Figure 7 provide the results of detailed analyses of clinical outcomes for patients with different degrees of hypoalbuminemia. Upon albumin supplementation, patients with higher albumin levels exhibited higher risks of mortality, shock, heart failure, and pulmonary edema in patients with albumin ≥1.0 g/dL. Mortality risk was significantly elevated in the 1.0–2.0 g/dL (HR, 1.221; 95% CI, 1.199–1.243), 2.0–3.0 g/dL (HR, 1.618; 95% CI, 1.596–1.641), and 3.0–3.5 g/dL groups (HR, 1.803; 95% CI, 1.775–1.831), while not significant in the 0.0–1.0 g/dL subgroup (HR, 1.050; 95% CI, 0.988–1.116).

Similarly, in shock outcomes, HRs were 1.074 (95% CI, 1.046–1.102), 1.324 (95% CI, 1.299–1.349), and 1.435 (1.405–1.465) in the 1.0–2.0, 2.0–3.0, and 3.0–3.5 g/dL groups, respectively, while no association was seen in the 0.0–1.0 g/dL group (HR, 0.990; 95% CI, 0.898–1.091, *p* = 0.8380).

For heart failure, HRs were 1.093 (95% CI, 1.076–1.110) and 1.148 (1.130–1.167) in the 2.0–3.0 and 3.0–3.5 g/dL groups, respectively, with no significant association below 2.0 g/dL.

Pulmonary edema followed a similar pattern, with significantly elevated HRs in the 1.0–2.0 g/dL (HR, 1.152; 95% CI, 1.108–1.197), 2.0–3.0 g/dL (HR, 1.477; 95% CI, 1.433–1.523), and 3.0–3.5 g/dL groups (HR, 1.477; 95% CI, 1.433–1.523), but not in the 0.0–1.0 g/dL group (HR, 1.479; 95% CI, 1.438–1.520). These results suggest a consistent association between albumin use and adverse outcomes in patients with low albumin levels.

### 3.3. Sensitivity Outcome

To determine the reliability of the results, sensitivity analysis was performed to assess 30-day mortality by using unadjusted and fully adjusted Cox proportional hazards models after a unified propensity score matching process that incorporated all relevant covariates, including demographics, comorbidities, laboratory data, and medications. The results remained robust and consistent with those of the main analysis, with no significant differences in 30-day mortality observed between the matched groups (Table 2).

## 4. Discussion

Our study addresses the long-standing controversy regarding the clinical importance of administering albumin to critically ill patients with hypoalbuminemia. In our study, the risk of 30-day mortality was higher in the patients with sepsis with hypoalbuminemia in the albumin infusion group than in the control group. In addition, compared with the control group, the albumin infusion group exhibited increased risks of shock, that is, of septic shock, cardiogenic shock, and hypovolemic shock. Furthermore, the risks of heart failure and pulmonary edema were higher in the albumin group than in the control group.

Albumin is a single polypeptide chain. It contributes to plasma colloid oncotic pressure; modulates apoptosis; helps maintain capillary permeability to macromolecules; carries molecules with low water solubility, such as hormones; and reduces inflammatory responses [6,16]. Albumin also has antioxidant, antiplatelet aggregation, and anticoagulant properties [6,17]. In addition, hypoalbuminemia is associated with poor clinical outcomes in acutely ill patients [6,18]. Thus, albumin supplementation is widely used to improve clinical outcomes in patients with critical illness.

A meta-analysis revealed that for every 10 mg/L decrease in the serum albumin concentration, the risk of mortality increased by 137% and that of morbidity increased by 89%, and the lengths of stay in the ICU and hospital were prolonged by 28% and 71%, respectively [19]. The albumin level can also serve as a prognostic marker of hypoalbuminemia; it can reflect levels of physiological stress due to inflammation resulting from disease or trauma [10]. A prospective randomized controlled study reports that giving albumin to critically ill patients with hypoalbuminemia may help support organ function by reducing fluid overload and enhancing tolerance to enteral nutrition [20]. Thus, albumin is commonly used to correct hypoalbuminemia. However, routine administration of albumin may not always be advantageous for treating hypoalbuminemia. According to current evidence, albumin supplementation should be employed only in specific scenarios [6,18], such as spontaneous bacterial peritonitis, hepatorenal syndrome, large-volume paracentesis, uncomplicated grade 2 and 3 ascites, severe acute pancreatitis, and Coronavirus Disease 2019 (COVID-19) infection [5,21,22,23,24,25].

Albumin administration may lead to increased mortality rates in hypovolemia, hypoproteinemia, and hypoalbuminemia [26,27]. Also, the SAFE study found that albumin and saline produced similar outcomes when used for fluid resuscitation in ICU patients [28]. In addition, albumin infusion did not lead to a reduction in fluid requirements, infection rate, or mortality in the ICU [10]. A cohort study reported on the adverse effects of albumin supplementation in hypoalbuminemia, including traumatic brain injury related to intracranial pressure [29]. In a meta-analysis of cohort studies, the treatment of hypoalbuminemia through albumin administration did not lead to an overall reduction in morbidity [19]. Administering human albumin might exacerbate the condition of critically ill patients through several mechanisms [27]. Several pathophysiological mechanisms have been proposed to explain these adverse effects. First, rapid volume replacement with albumin may lead to cardiac decompensation due to increased volume retention [27]. Second, in patients with increased capillary permeability or capillary leak syndrome, albumin administration may exacerbate or cause pulmonary edema as a result of albumin and water crossing the capillary membrane, which may compromise tissue oxygenation and result in multiorgan failure [30]. Finally, the antihemostatic and platelet-lowering properties of albumin may increase the volume of blood loss in patients after surgery or those with trauma [31]. These mechanisms may explain the main finding of our study.

As for inflammation, it is linked to hypoalbuminemia; it can increase capillary permeability, leading to serum albumin crossing the capillary membrane, which may contribute to increases in the volume of the interstitial space. Thus, hypoalbuminemia may reflect inflammatory status [10]. Notably, sepsis is a systemic inflammatory response to infection, and severe sepsis is considered to be sepsis complicated by acute organ dysfunction [11,32]. Because it is a severe infection, sepsis is frequently accompanied by hypoalbuminemia [12]. In a randomized control study, Dubois et al. demonstrated that based on SOFA scores, albumin administration in critically ill patients with hypoalbuminemia may improve organ function [20,33], particularly in the respiratory, cardiovascular, and central nervous systems [34]. In addition, the SAFE study suggested that administering albumin, as compared to administering saline, to patients with severe sepsis did not worsen organ function and might have reduced the risk of death. When the albumin and saline groups were compared, the relative risk of mortality was 0.87 for patients with severe sepsis and 1.05 for those without severe sepsis [35]. However, in the ALBIOS trial, Caironi et al. analyzed 1818 patients with severe sepsis in the ICU and reported that albumin replacement did not improve 28- and 90-day survival rates, although the patients who underwent albumin replacement exhibited high serum albumin levels, high mean arterial pressure, and low net fluid balance [12].

Given the aforementioned inconsistent findings, the current study used the TriNetX database to investigate the effects of albumin supplementation in patients with sepsis with hypoalbuminemia. Our results align with the ALBIOS trial in that albumin supplementation did not improve short-term mortality.

This study conducted subgroup analyses by albumin levels to examine clinical outcomes in patients with hypoalbuminemia, who were divided into the following subgroups: 0–1, 1–2, 2–3, and 3–3.5 g/dL. The analyses revealed increased HRs with increased albumin levels. In addition, the analyses revealed that patients with albumin levels of 3.0–3.5 g/dL exhibited the highest HR, followed by those with albumin levels of 2.0–3.0 and 1.0–2.0 g/dL. By contrast, mortality was not increased in patients with severe hypoalbuminemia, as indicated by albumin levels of 0.0–1.0 g/dL. These results suggest that albumin supplementation is associated with increased mortality risk in patients with higher albumin levels; however, albumin supplementation has no significant impact on patients with severe hypoalbuminemia. This trend was consistent with the findings for shock, pulmonary edema, and heart failure in this study. That is, under albumin supplementation, patients with higher albumin levels tended to exhibit higher risks of mortality, shock, heart failure, and pulmonary edema, whereas albumin supplementation did not exert any significant influence on patients with severe hypoalbuminemia (0.0–1.0 g/dL). Thus, caution should be exercised in administering albumin in patients with varying albumin levels at baseline. The observed association between albumin administration and increased adverse outcomes may be influenced by several factors. First, patients with hypoalbuminemia are more susceptible to fluid overload from albumin infusion, which may exacerbate pulmonary or cardiovascular complications. Second, our dataset lacks direct indicators of disease severity, which limits our ability to fully adjust for baseline illness acuity and introduces the possibility of residual confounding. Third, the timing of albumin administration was not captured in the database. These could lead to treatment bias and affect outcomes differently across clinical contexts. These factors collectively underscore the complexity of interpreting outcomes associated with albumin use and highlight the need for prospective studies with more granular clinical data.

A meta-analysis on the albumin intervention for hypoalbuminemia in patients with acute illness demonstrated a survival benefit in the albumin group when the albumin levels exceeded 3 g/dL [19]. This benefit may be attributable to several factors associated with higher albumin levels, including a lower incidence of pneumonia [36] and greater relief from hypotension [37].

Given the inconsistency with some previous studies, our findings demonstrate the need for future large-scale randomized controlled trials to confirm these results and clarify the clinical role of albumin supplementation in this patient population. Further investigation is warranted into the mechanisms underlying the higher mortality risk associated with higher albumin levels in our study.

## 5. Limitation and Strength

First, we were unable to access individual-level data or obtain the precise number of patients at risk at each time point from the TriNetX database. Such information cannot be obtained—primarily because of concerns related to patient privacy and adherence to data-sharing regulations—and users are provided with survival estimates at fixed intervals (e.g., at 7, 14, or 21 days) instead of estimates for every event time. Therefore, creating a precise “at-risk” table was unfeasible in this study; doing so would require knowledge of the number of participants under observation at each time point. This limitation may hinder detailed time-to-event analyses and should be considered in interpreting the current findings. Second, we excluded patients with any form of shock, which limits the generalizability of the results to non-critically septic patients. Third, our retrospective cohort study design may introduce various biases, including selection bias and bias related to unmeasured confounders. Although we employed PSM to minimize disparities in baseline variables between the groups, all residual confounders could not be eliminated. Patients who received albumin therapy may have been more severely ill at baseline. As such, albumin use could be a marker of illness severity rather than a direct cause of increased mortality. Therefore, attributing mortality solely to albumin administration remains uncertain. Fourth, in this large-scale cohort, comorbidities were included at a broad disease category. This approach was chosen to enhance confounding control given the study’s scale. However, we acknowledge that such broad classifications may encompass heterogeneous clinical entities, which limits the granularity of interpretation and may reduce clinical specificity in the matched analysis. Additionally, we did not consider several potentially crucial factors—such as patient performance status and treatment adherence—which may affect clinical outcomes. Last, the dosage and timing of albumin administration may significantly influence clinical outcomes. However, within the TriNetX database, we were only able to identify whether patients had received albumin supplementation, without access to the exact timing or dosage of administration.

Despite these limitations, this study has several strengths. By using the global TriNetX network, which integrates de-identified data from multiple healthcare systems, we accessed extensive, longitudinal data on patient diagnoses, treatments, laboratory information, and clinical outcomes. The use of this vast data infrastructure not only enabled reliable time-to-event analyses but also enhanced the external validity of our conclusions. Furthermore, our comprehensive subgroup analyses by different albumin levels and the analyses of the effects of albumin infusion at each albumin level indicated favorable prognoses in patients who did not receive albumin infusion. These nuanced findings have vital clinical implications. That is, refraining from albumin infusion may result in more favorable outcomes in patients with various albumin levels.

## 6. Conclusions

In patients with sepsis and hypoalbuminemia, the risks of 30-day mortality, shock, heart failure, and pulmonary edema were significantly higher than those of the control group. These findings suggest that routine albumin administration was associated with clinically unfavorable outcomes and should be used with caution, particularly in patients with higher baseline albumin levels. These findings conflict with some prior evidence, and further large-scale randomized controlled studies are needed to validate our findings.

## Figures and Tables

**Table 1:** Baseline characteristics of the cohort (before and after the propensity score matching).

	Before Matching			After Matching		
Clinical Characteristics	Albumin Group (*n* = 188,365)	Non-Albumin Group (*n* = 959,068)	Std Diff	Albumin Group (*n* = 188,365)	Non-Albumin Group (*n* = 188,365)	Std Diff
Age at index						
Mean ± SD	67.8 ± 15.7	68.6 ± 17.2	0.049	67.8 ± 15.7	67.6 ± 16.7	0.015
Gender						
Female	81,722 (43.4%)	445,488 (46.5%)	0.062	81,722 (43.4%)	80,666 (42.8%)	0.011
Male	99,107 (52.6%)	484,002 (50.5%)	0.043	99,107 (52.6%)	99,750 (53.0%)	0.007
Race						
White	113,392 (60.2%)	581,668 (60.6%)	0.009	113,392 (60.2%)	113,736 (60.4%)	0.004
Black or African American	30,850 (16.3%)	157,990 (16.5%)	0.003	30,850 (16.3%)	29,322 (15.6%)	0.022
Asian	15,313 (8.1%)	55,469 (5.8%)	0.092	15,313 (8.1%)	16,647 (8.8%)	0.025
American Indian or Alaska Native	602 (0.3%)	2749 (0.3%)	0.006	602 (0.3%)	639 (0.3%)	0.003
Native Hawaiian or Other Pacific Islander	2052 (1.1%)	8668 (0.9%)	0.019	2052 (1.1%)	1980 (1.1%)	0.004
Other races	7685 (4.1%)	36,185 (3.8%)	0.016	7685 (4.1%)	7653 (4.1%)	<0.001
Unknown Ethnicity	30,758 (16.3%)	203,373 (21.2%)	0.125	30,758 (16.3%)	29,730 (15.8%)	0.015
Comorbidities						
Endocrine, nutritional and metabolic diseases	170,936 (90.7%)	734,633 (76.6%)	0.390	170,936 (90.7%)	167,920 (89.1%)	0.053
Disease of the digestive system	157,787 (83.8%)	620,842 (64.7%)	0.446	157,787 (83.8%)	158,442 (84.1%)	0.009
Disease of the circulatory system	168,750 (89.6%)	725,709 (75.7%)	0.374	168,750 (89.6%)	166,680 (88.5%)	0.035
Disease of the genitourinary system	156,778 (83.2%)	629,362 (65.6%)	0.412	156,778 (83.2%)	155,279 (82.4%)	0.021
Disease of the respiratory system	151,860 (80.6%)	586,127 (61.1%)	0.440	151,860 (80.6%)	150,787 (80.1%)	0.014
Diseases of the musculoskeletal system and connective tissue	130,595 (69.3%)	593,420 (61.9%)	0.157	130,595 (69.3%)	128,866 (68.4%)	0.020
Certain infectious and parasitic disease	169,408 (89.9%)	562,844 (58.7%)	0.766	169,408 (89.9%)	167,730 (89.0%)	0.029
Disease of the nervous system	135,290 (71.8%)	541,817 (56.5%)	0.324	135,290 (71.8%)	134,285 (71.3%)	0.012
Mental, Behavioral and Neurodevelopmental disorders	115,170 (61.1%)	492,546 (51.4%)	0.200	115,170 (61.1%)	114,291 (60.7%)	0.010
Neoplasms	85,052 (45.2%)	355,606 (37.1%)	0.165	85,052 (45.2%)	85,085 (45.2%)	<0.001
External causes of morbidity	78,730 (41.8%)	305,7635 (31.9%)	0.206	78,730 (41.8%)	78,703 (41.8%)	<0.001
Congenital malformations, deformations and chromosomal abnormalities	22,924 (12.2%)	83,702 (8.7%)	0.113	22,924 (12.2%)	22,849 (12.1%)	0.001
Medication						
Central nervous system medications	178,513 (94.8%)	797,469 (83.2%)	0.377	178,513 (94.8%)	175,837 (93.3%)	0.060
Respiratory tract medications	177,141 (94.0%)	757,689 (79.0%)	0.452	177,141 (94.0%)	174,945 (92.87%)	0.047
Gastrointestinal medications	176,099 (93.5%)	753,901 (78.6%)	0.440	176,099 (93.5%)	174,072 (92.4%)	0.042
Antimicrobials	173,956 (92.4%)	739,684 (77.1%)	0.433	173,956 (92.4%)	171,382 (91.0%)	0.050
Cardiovascular medications	172,644 (91.7%)	743,815 (77.6%)	0.398	172,644 (91.7%)	171,190 (90.9%)	0.027
Hormones/synthetics/modifiers	170,538 (90.5%)	717,420 (74.8%)	0.425	170,538 (90.5%)	168,895 (89.7%)	0.029
Genitourinary medications	157,804 (83.8%)	626,166 (65.3%)	0.434	157,804 (83.8%)	157,741 (83.7%)	<0.001
Musculoskeletal medications	134,090 (71.2%)	549,287 (57.3%)	0.293	134,090 (71.2%)	134,825 (71.6%)	0.009
Antiparasitics	10,564 (5.6%)	38,241 (4.0%)	0.076	10,564 (5.6%)	10,377 (5.6%)	0.002
Antineoplastics	33,939 (18.0%)	135,261 (14.1%)	0.107	33,939 (18.0%)	33,991 (18.0%)	<0.001
Laboratory						
Sodium [Moles/volume] in Serum, Plasma or Blood	137 ± 5.82	137 ± 4.77	0.161	137 ± 5.82	137 ± 4.91	0.116
Potassium [Moles/volume] in Serum, Plasma or Blood	4.04 ± 0.677	4.06 ± 0.592	0.036	4.04 ± 0.677	4.06 ± 0.605	0.033
Creatinine [Mass/volume] in Serum, Plasma or Blood	1.94 ± 2.64	1.48 ± 3.86	0.151	1.94 ± 2.64	1.58 ± 3.23	0.120
Hematocrit [Volume Fraction] of Blood	30.9 ± 7.46	33.9 ± 8.73	0.372	30.9 ± 7.46	34 ± 7.64	0.415
Neutrophils [#/volume] in Blood	347 ± 2242	142 ± 1273	0.113	347 ± 2242	276 ± 1871	0.034
Lymphocytes/100 leukocytes in Blood	13.2 ± 11.8	16.7 ± 12.5	0.286	13.2 ± 11.8	16 ± 12.5	0.227
Monocytes/100 leukocytes in Blood	7.21 ± 4.57	7.88 ± 4.22	0.153	7.21 ± 4.57	7.79 ± 4.34	0.130
Eosinophils/100 leukocytes in Blood	1.57 ± 2.63	1.84 ± 2.6	0.101	1.57 ± 2.63	1.8 ± 2.67	0.083
Basophils/100 leukocytes in Blood	0.38 ± 0.546	0.445 ± 0.567	0.116	0.38 ± 0.546	0.432 ± 0.53	0.097

Table 1 shows well-matched baseline characteristics after 1:1 propensity score matching. Std diff, Standardized Difference. SD, standard deviation.

**Table 2:** Sensitivity test with different covariate groups under PSM.

Clinical Outcomes	After Matching			
	Model 1		Model 2	
	HR (95% CI)	*p* Value	HR (95% CI)	*p* Value
Overall Survival	2.162 (2.134, 2.192)	<0.0001	1.800 (1.774, 1.827)	<0.0001

Models for confounding adjusting: Model 1 presents the unadjusted Cox model after PSM. Model 2 presents the fully adjusted Cox model after PSM, incorporating all covariates used for matching. HR, hazard ratio. CI, confidence interval.

## Data Availability

The data that support the findings of this study were obtained from the TriNetX Research Network. All search criteria (ICD-10 codes, RxNorm medications, time windows, and exclusion rules), the complete analytic workflow, and the aggregated, de-identified output generated by TriNetX are provided in Supplementary Material S1.

## Supplementary Materials

The following supporting information can be downloaded at: https://www.mdpi.com/article/10.3390/jcm14134790/s1, Supplementary Material S1: Compare Outcomes Analysis.
