# Supplementary material for: The Efficacy of Albumin Infusion in Septic Patients with Hypoalbuminemia: An International Retrospective Observational Study
Source: J Clin Med. 2025 Jul 7;14(13):4790. doi: 10.3390/jcm14134790 (PMC12251147; doi:10.3390/jcm14134790)
Supplement: Supplementary file 1 [file jcm-14-04790-s001.zip › jcm-3697413-supplementary.pdf]

TriNetX Explore real world, real-time global data

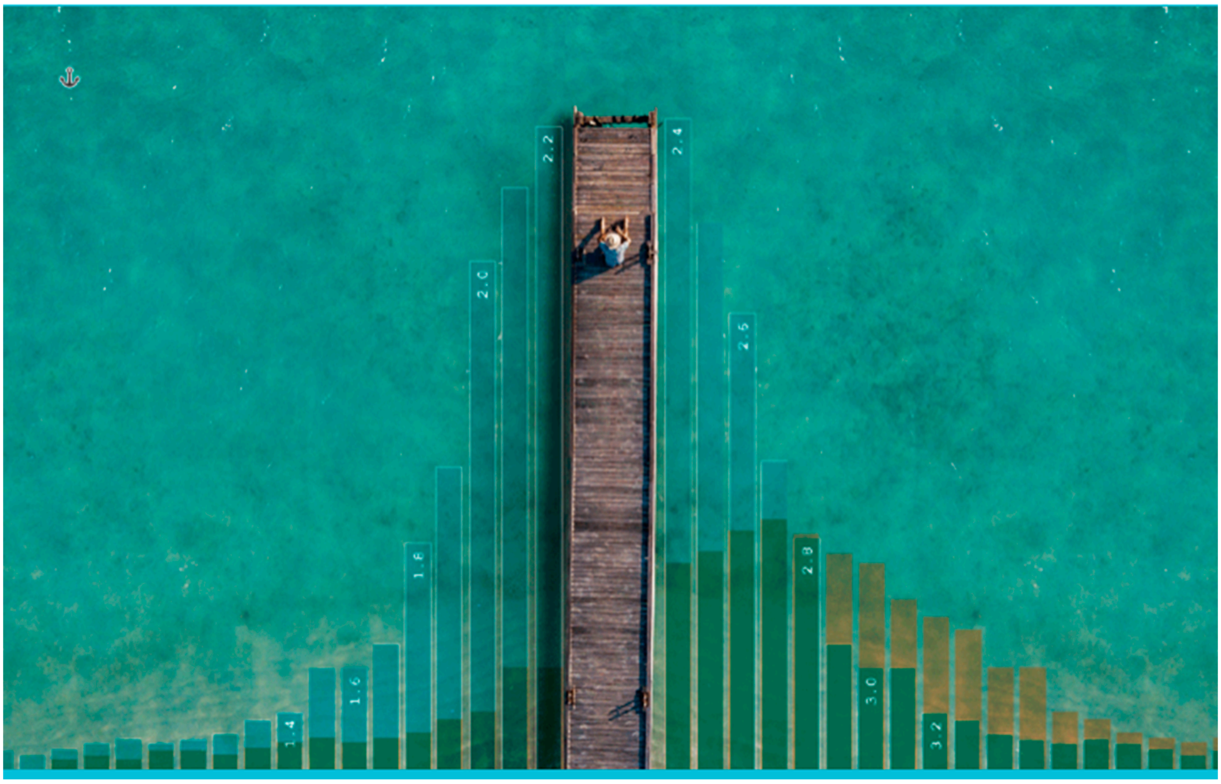

Supplementary Material 1

Compare Outcomes Analysis

Created by TriNetX on Mar 14, 2025, 05:22:55 UTC

Introduction

TriNetX is the global federated health research network providing access to electronic medical records (diagnoses, procedures, medications, laboratory values, genomic information) across large healthcare organizations (HCOs). This report was run on the set of HCOs grouped into a network called Global Collaborative Network. This network included 141 HCO(s).

This report describes a Compare Outcomes Analysis, named Main result-age restriction, generated by the TriNetX platform on Mar 14, 2025, 05:22:55 UTC. This analysis compared the outcomes of two cohorts: Cohort A (237,048 patients) named main with albumin-age restriction and Cohort B (1,193,103 patients) named main without albumin-age restriction.

This analysis was run by Hsin-Yu LIU (jill10230433@gmail.com) and downloaded by Shih-Sen Lin (fivewood103@gmail.com).

Methods

The analysis process includes two main steps: 1) Defining the cohorts through query criteria; 2) Setting up and running the analysis. Setting up the analysis requires definitions for the index event, outcomes criteria, and the time frame. Compare outcomes supports four analyses: Measures of Association, Survival, Number of Instances and Lab result distribution. These analyses have additional options that are listed in the Outcomes Definitions and Analyses Specifications section below. Furthermore, characteristics of the cohorts that are balanced using propensity score matching are also included in the Propensity Score Matching section.

Cohorts definition

This section lists all terms used in the definitions of the two cohorts.

Query Criteria for Cohort 1 (query name: main with albumin-age restriction)

This query was run on the network Global Collaborative Network with 141 HCO(s) queried and 141 HCO(s) responded. A total of 91 provider(s) responded with patients. The final cohort included 237,048 patients who matched the query criteria listed in the table below. For the text representation of the query criteria please see Appendix A.

**Ungrouped terms**					
	must have		demographics	Age	Age (at least 18 years (most recent occurrence))
**Group 1**					
	**Group 1A Sepsis with hypoalbuminemia**				
	must have		diagnosis	UMLS:ICD10CM:A41.9	Sepsis, unspecified organism
		and	laboratory	TNX:9045	Albumin [Mass/volume] in Serum, Plasma or Blood (between 0.00 and 3.50 g/dL)
	cannot have		diagnosis	UMLS:ICD10CM:R57.9	Shock, unspecified
	date constraint		The terms in this group occurred on or after Apr 1, 2014		
	event relationship		Any instance of albumin infusion occurred on or after any instance of Sepsis with hypoalbuminemia		
	**Group 1B albumin infusion**				

must have	any of	medication	NLM:RXNORM:317176	albumin,aggregated
		medication	NLM:RXNORM:828529	albumin human, USP
		medication	NLM:RXNORM:1370439	albumin microspheres, human
		medication	NLM:RXNORM:1426381	serum albumin, bovine
		medication	NLM:RXNORM:314301	albumin,iodinated I-131 serum
		medication	NLM:RXNORM:OMOP4819585	albumin tannate

Query Criteria for Cohort 2 (query name: main without albumin-age restriction)

This query was run on the network Global Collaborative Network with 141 HCO(s) queried and 141 HCO(s) responded. A total of 113 provider(s) responded with patients. The final cohort included 1,193,103 patients who matched the query criteria listed in the table below.

**Ungrouped terms**				
must have		demographics	Age	Age (at least 18 years (most recent occurrence))
**Group 1**				
**Group 1A Sepsis with hypoalbuminemia**				
must have		diagnosis	UMLS:ICD10CM:A41.9	Sepsis, unspecified organism
	and	laboratory	TNX:9045	Albumin [Mass/volume] in Serum, Plasma or Blood (between 0.00 and 3.50 g/dL)
cannot have		diagnosis	UMLS:ICD10CM:R57.9	Shock, unspecified
date constraint		The terms in this group occurred on or after Apr 1, 2014		
event relationship		Any instance of albumin infusion occurred on or after any instance of Sepsis with hypoalbuminemia		
**Group 1B albumin infusion**				
cannot have	all of	medication	NLM:RXNORM:317176	albumin,aggregated
		medication	NLM:RXNORM:1370439	albumin microspheres, human
		medication	NLM:RXNORM:OMOP4819585	albumin tannate
		medication	NLM:RXNORM:314301	albumin,iodinated I-131 serum
		medication	NLM:RXNORM:828529	albumin human, USP
		medication	NLM:RXNORM:1426381	serum albumin, bovine

Analysis Setup

This section contains the Index Event and Time Window definitions and a list of selected outcomes and the analyses.

Index Event & Time Window Definitions

The index event defines the point in time when each patient in the cohort enters the analysis. To define an index event for the cohort, one or more criteria for the cohort must be selected. The index date for each patient within a cohort is the day on which the patient first met the selected criteria for the cohort (listed in the table below).

As the index event defines the earliest time point after which outcomes are analyzed, the time window defines the duration during which outcomes are analyzed. The time window can start on the same day as the index event or at any specified time interval after the index event. The time window can end any time after the start date. Outcomes are defined as diagnoses, medications, procedures, or laboratory values that happened in the time window starting after the first occurrence of the index event.

Time Window Used in this Analysis

This analysis included outcomes that occurred in the time window that started 1 day after the first occurrence of the index event and ended 30 days after the first occurrence of the index event.

The index event only includes events that occurred up to 20 years ago. Patients whose index event occurred 20 years or more ago are excluded. In this analysis, 0 patients in Cohort 1 and 0 patients in Cohort 2 were excluded because they met the index event more than 20 years ago.

Index Events Used in this Analysis

Index events for the Compare Outcomes analysis were derived from the cohort definitions. Index events were defined separately for each cohort and were based on the criteria used in the original cohort definition. Please see Appendix B for the text representation of the index event definition.

The index event for Cohort 1 (query name: main with albumin-age restriction) was defined as the following:

**Group 1**				
**Group 1A Sepsis with hypoalbuminemia**				
must have		diagnosis	UMLS:ICD10CM:A41.9	Sepsis, unspecified organism
	and	laboratory	TNX:9045	Albumin [Mass/volume] in Serum, Plasma or Blood (between 0.00 and 3.50 g/dL)
cannot have		diagnosis	UMLS:ICD10CM:R57.9	Shock, unspecified
date constraint		The terms in this group occurred on or after Apr 1, 2014		
event relationship		Any instance of albumin infusion occurred on or after any instance of Sepsis with hypoalbuminemia		
**Group 1B albumin infusion**				
must have	any of	medication	NLM:RXNORM:317176	albumin,aggregated
		medication	NLM:RXNORM:828529	albumin human, USP
		medication	NLM:RXNORM:1370439	albumin microspheres, human
		medication	NLM:RXNORM:1426381	serum albumin, bovine
		medication	NLM:RXNORM:314301	albumin,iodinated I-131 serum
		medication	NLM:RXNORM:OMOP4819585	albumin tannate

The index event for Cohort 2 (query name: main without albumin-age restriction) was defined as the following:

**Group 1**				
**Group 1A Sepsis with hypoalbuminemia**				
must have		diagnosis	UMLS:ICD10CM:A41.9	Sepsis, unspecified organism
	and	laboratory	TNX:9045	Albumin [Mass/volume] in Serum, Plasma or Blood (between 0.00 and 3.50 g/dL)
cannot have		diagnosis	UMLS:ICD10CM:R57.9	Shock, unspecified
date constraint		The terms in this group occurred on or after Apr 1, 2014		
event relationship		Any instance of albumin infusion occurred on or after any instance of Sepsis with hypoalbuminemia		
**Group 1B albumin infusion**				
cannot have	all of	medication	NLM:RXNORM:317176	albumin,aggregated
		medication	NLM:RXNORM:1370439	albumin microspheres, human
		medication	NLM:RXNORM:OMOP4819585	albumin tannate
		medication	NLM:RXNORM:314301	albumin,iodinated I-131 serum
		medication	NLM:RXNORM:828529	albumin human, USP
		medication	NLM:RXNORM:1426381	serum albumin, bovine

Analyses Specifications

The Compare Outcomes Analytic supports four types of analyses: Measure of Association, Survival, Number of Instances, and Lab result distribution. The first three analyses support the “exclude patients with outcomes prior to the window” setting. This option can exclude patients from the analysis if they are not at risk for an outcome (e.g., if the outcome is a chronic disease). When "exclude patients with the outcome prior to the time window" is not checked, all patients in the cohort are included in the analysis, regardless of whether they had the outcome prior to the time window. When "exclude patients with the outcome prior to the time window" is checked, patients are excluded from the analysis if their record includes the outcome prior to the beginning of the time window. This selection will exclude all patients with the outcome prior to the index event. If the start of the time window for the analysis falls some days after the index event, patients will also be excluded if they have the outcome between the index event and the start of the time window.

Measure of Association Analysis

The Measure of Association Analysis calculates and compares the fraction of patients with the selected outcome. The output summary includes: Patients in each Cohort (count of patients meeting query criteria); Patients with Outcome in each Cohort (of the patients in the cohort, count of patients that had the outcome in the time window); and Risk (the fraction of patients in the cohort that have the outcome in the time window, i.e. Patients with Outcome / Patients in Cohort). In addition, Risk Difference (the difference in the risks in Cohort 1 and Cohort 2), Risk Ratio (the ratio of the risks in Cohort 1 and Cohort 2), and Odds Ratio (the ratio of the odds in Cohort 1 and Cohort 2). The bar chart shows the risk of the outcome for the both cohorts.

Survival Analysis

The Kaplan-Meier Analysis estimates probability of the outcome at a respective time interval (daily time interval is used in this analysis). In order to account for the patients who exited the cohort during the analysis period, and therefore should not be included in the analysis, censoring is applied. In this analysis, patients are removed from the analysis (censored) after the last fact in their record.

The output summary includes: Patients in each Cohort (count of patients meeting query criteria); Patients with Outcome (of the patients in the cohort, count of patients that had the outcome in the time window); Median Survival (the number of days when the survival drops below 50%; the “-” indicates that survival does not drop below 50% during the time window); and Survival Probability at End of Time Window (the % survival at the end of the time window). In addition, Log-Rank test, Hazard Ratio and test for Proportionality.

Number of Instances Analysis

The Number of Instances Analysis calculates how many times the outcome occurred in the time window. This analysis includes two additional settings: include patients with zero instances; the definition of an instance.

Selecting to exclude patients with zero instances will remove these patients from the calculations for mean number of instances, standard deviation, or median. The histogram showing the distribution of patients by number of instances will not contain a bar for zero. Alternatively, by selecting to include patients with zero instances, the mean, standard deviation, and median for number of instances will reflect these patients. The histogram will contain a bar for zero patients.

The definition of an instance affects how counts are analyzed. By selecting Date, each calendar date on which any of the terms selected in the outcome are recorded will represent one instance. For example, if the outcome is “Med A or Med B,” and a patient has “Med A” on January 3, then both medications on January 4, then “Med B” on January 6, then that patient is considered to have three instances– January 3, January 4, and January 6. Note that if an outcome occurs across several dates (e.g. Visit: inpatient encounter), then only the start date is tracked for the purpose of counting instances. A patient who begins at stay on January 1, ends that stay on January 3, begins another stay on January 10, and ends that stay on January 15, is considered to have two instances of the outcome.

Selecting Visit as an Instance will count any visit that includes the outcome as one instance, regardless of how many times it occurred. For instance, consider a patient administered an analgesic on each of the three days that make up an inpatient stay following some index event. If analgesic is an outcome, these three administrations will represent only one instance, because all three are associated with the same visit.

The output summary includes: Patients in Cohort (count of patients meeting query criteria); Patients with Outcome (of the patients in the cohort, count of patients that had the outcome in the time window); Mean (mean of the counts); Standard Deviation (standard deviation of the counts); Median (median of the counts); and Median (1+ instances) when patients with zero instances included in the analysis. In addition, T-Test statistics testing for the difference between the cohorts is included.

Laboratory Results Analysis

Lab Results can be included in the analysis only for the outcomes that are labs. Only the most recent lab values in the time window are included. For the lab results that are numeric, the outcome summary includes: Patients in Cohort (count of patients meeting query criteria); Patients with Outcome (of the patients in the cohort, count of patients that had the outcome in the time window); Mean (mean of the counts); and Standard Deviation (the standard deviation for lab values across patients in the cohort). In addition, T-Test statistics testing for the difference between the cohorts is included.

For the non-numeric lab results, three values are reported: counts of Negative; Positives; and Unknowns. The counts are represented in the bar chart as percentages of the total counts.

Outcome Definitions

Table below outlines the definitions for each outcome and the analysis specifications. For outcome definitions consisting of more than one term, at least one term must match. Please see Appendix C for the text representation of the outcome definitions.

**Mortality**		
**Outcome definition**		
Demographics	Deceased	Deceased
Diagnosis	UMLS:ICD10CM:R99	Ill-defined and unknown cause of mortality
**Settings for the performed analyses**		
Risk analysis		including patients with outcome prior to the time window
Kaplan - Meier survival analysis		including patients with outcome prior to the time window
**Shock**		
**Outcome definition**		
Diagnosis	UMLS:ICD10CM:R57.9	Shock, unspecified
Diagnosis	UMLS:ICD10CM:R57	Shock, not elsewhere classified
Diagnosis	UMLS:ICD10CM:R65.21	Severe sepsis with septic shock
Diagnosis	UMLS:ICD10CM:R57.1	Hypovolemic shock
Diagnosis	UMLS:ICD10CM:R57.0	Cardiogenic shock
**Settings for the performed analyses**		

Risk analysis		including patients with outcome prior to the time window
Kaplan - Meier survival analysis		including patients with outcome prior to the time window
**Septic shock**		
**Outcome definition**		
Diagnosis	UMLS:ICD10CM:R65.21	Severe sepsis with septic shock
**Settings for the performed analyses**		
Risk analysis		including patients with outcome prior to the time window
Kaplan - Meier survival analysis		including patients with outcome prior to the time window
**Hypovolemic shock**		
**Outcome definition**		
Diagnosis	UMLS:ICD10CM:R57.1	Hypovolemic shock
**Settings for the performed analyses**		
Kaplan - Meier survival analysis		including patients with outcome prior to the time window
Risk analysis		including patients with outcome prior to the time window
**Cardiogenic shock**		
**Outcome definition**		
Diagnosis	UMLS:ICD10CM:R57.0	Cardiogenic shock
**Settings for the performed analyses**		
Risk analysis		including patients with outcome prior to the time window
Kaplan - Meier survival analysis		including patients with outcome prior to the time window
**Heart failure**		
**Outcome definition**		
Diagnosis	UMLS:ICD10CM:I50	Heart failure
Diagnosis	UMLS:ICD10CM:I50.1	Left ventricular failure, unspecified
Diagnosis	UMLS:ICD10CM:I50.9	Heart failure, unspecified
Diagnosis	UMLS:ICD10CM:I50.30	Unspecified diastolic (congestive) heart failure
Diagnosis	UMLS:ICD10CM:I50.20	Unspecified systolic (congestive) heart failure
**Settings for the performed analyses**		
Risk analysis		including patients with outcome prior to the time window
Kaplan - Meier survival analysis		including patients with outcome prior to the time window
**Pulmonary edema**		
**Outcome definition**		
Diagnosis	UMLS:ICD10CM:J81	Pulmonary edema
Diagnosis	UMLS:ICD10CM:J81.0	Acute pulmonary edema
**Settings for the performed analyses**		
Kaplan - Meier survival analysis		including patients with outcome prior to the time window
Risk analysis		including patients with outcome prior to the time window

Propensity Score Matching

Propensity score matching was performed on all listed characteristics. Characteristics of the cohorts before and after matching are summarized in the table below.

**Cohort 1 and cohort 2 patient count before and after propensity score matching**		
Cohort	Patient count before matching	Patient count after matching
1 - main with albumin-age restriction	188,365	188,365
2 - main without albumin-age restriction	959,068	188,365
**Propensity score density function - Before and after matching (cohort 1 - purple, cohort 2 - green)**		

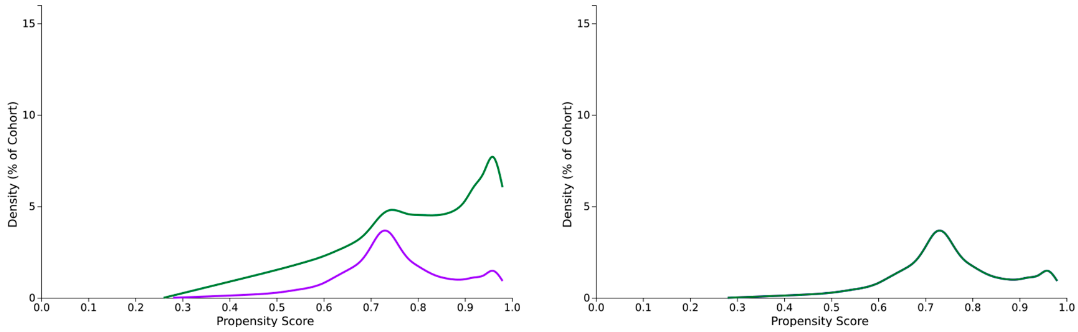

Cohort 1 (N = 188,365) and cohort 2 (N = 959,068) characteristics before propensity score matching							
**Demographics**							
Cohort			Mean ± SD	Patients	% of Cohort	P-Value	Std diff.
1<br>2	Age	Current Age	67.8 +/- 15.7<br>68.6 +/- 17.2	188,365<br>959,068	100%<br>100%	<0.001	0.049
1<br>2	AI	Age at Index	63.6 +/- 16.1<br>64.4 +/- 17.7	188,365<br>959,068	100%<br>100%	<0.001	0.050
1<br>2	F	Female		81,722<br>445,488	43.4%<br>46.5%	<0.001	0.062
1<br>2	2054-5	Black or African American		30,850<br>157,990	16.4%<br>16.5%	0.307	0.003
1<br>2	M	Male		99,107<br>484,002	52.6%<br>50.5%	<0.001	0.043
1<br>2	2106-3	White		113,392<br>581,668	60.2%<br>60.6%	<0.001	0.009
1<br>2	1002-5	American Indian or Alaska Native		602<br>2,749	0.3%<br>0.3%	0.015	0.006
1<br>2	UNK	Unknown Race		18,471<br>116,339	9.8%<br>12.1%	<0.001	0.074
1<br>2	2076-8	Native Hawaiian or Other Pacific Islander		2,052<br>8,668	1.1%<br>0.9%	<0.001	0.019
1<br>2	UN	Unknown Ethnicity		30,758<br>203,373	16.3%<br>21.2%	<0.001	0.125
1<br>2	2186-5	Not Hispanic or Latino		139,530<br>673,562	74.1%<br>70.2%	<0.001	0.086
1<br>2	2135-2	Hispanic or Latino		18,077<br>82,133	9.6%<br>8.6%	<0.001	0.036
1<br>2	2131-1	Other Race		7,685<br>36,185	4.1%<br>3.8%	<0.001	0.016
1<br>2	2028-9	Asian		15,313<br>55,469	8.1%<br>5.8%	<0.001	0.092
**Diagnosis**							
Cohort			Mean ± SD	Patients	% of Cohort	P-Value	Std diff.
1<br>2	E00-E89	Endocrine, nutritional and metabolic diseases		170,936<br>734,633	90.7%<br>76.6%	<0.001	0.390
1<br>2	K00-K95	Diseases of the digestive system		157,787<br>620,842	83.8%<br>64.7%	<0.001	0.446
1<br>2	I00-I99	Diseases of the circulatory system		168,750<br>725,709	89.6%<br>75.7%	<0.001	0.374

1<br>2	N00-N99	Diseases of the genitourinary system		156,778<br>629,362	83.2%<br>65.6%	<0.001	0.412
1<br>2	J00-J99	Diseases of the respiratory system		151,860<br>586,127	80.6%<br>61.1%	<0.001	0.440
1<br>2	M00-M99	Diseases of the musculoskeletal system and connective tissue		130,595<br>593,420	69.3%<br>61.9%	<0.001	0.157
1<br>2	A00-B99	Certain infectious and parasitic diseases		169,408<br>562,844	89.9%<br>58.7%	<0.001	0.766
1<br>2	G00-G99	Diseases of the nervous system		135,290<br>541,817	71.8%<br>56.5%	<0.001	0.324
1<br>2	F01-F99	Mental, Behavioral and Neurodevelopmental disorders		115,170<br>492,546	61.1%<br>51.4%	<0.001	0.198
1<br>2	C00-D49	Neoplasms		85,052<br>355,606	45.2%<br>37.1%	<0.001	0.165
1<br>2	V00-Y99	External causes of morbidity		78,730<br>305,763	41.8%<br>31.9%	<0.001	0.207
1<br>2	Q00-Q99	Congenital malformations, deformations and chromosomal abnormalities		22,924<br>83,702	12.2%<br>8.7%	<0.001	0.113
**Medication**							
Cohort			Mean ± SD	Patients	% of Cohort	P-Value	Std diff.
1<br>2	CN000	CENTRAL NERVOUS SYSTEM MEDICATIONS		178,513<br>797,469	94.8%<br>83.2%	<0.001	0.377
1<br>2	RE000	RESPIRATORY TRACT MEDICATIONS		177,141<br>757,689	94.0%<br>79.0%	<0.001	0.451
1<br>2	GA000	GASTROINTESTINAL MEDICATIONS		176,099<br>753,901	93.5%<br>78.6%	<0.001	0.440
1<br>2	AM000	ANTIMICROBIALS		173,956<br>739,684	92.4%<br>77.1%	<0.001	0.433
1<br>2	CV000	CARDIOVASCULAR MEDICATIONS		172,644<br>743,815	91.7%<br>77.6%	<0.001	0.398
1<br>2	HS000	HORMONES/SYNTHETICS/ MODIFIERS		170,538<br>717,420	90.5%<br>74.8%	<0.001	0.425
1<br>2	GU000	GENITOURINARY MEDICATIONS		157,804<br>626,166	83.8%<br>65.3%	<0.001	0.434
1<br>2	MS000	MUSCULOSKELETAL MEDICATIONS		134,090<br>549,287	71.2%<br>57.3%	<0.001	0.293
1<br>2	AP000	ANTIPARASITICS		10,564<br>38,241	5.6%<br>4.0%	<0.001	0.076
1<br>2	AN000	ANTINEOPLASTICS		33,939<br>135,261	18.0%<br>14.1%	<0.001	0.107
**Laboratory**							
Cohort			Mean ± SD	Patients	% of Cohort	P-Value	Std diff.
1<br>2	9029	Sodium [Moles/volume] in Serum, Plasma or Blood	136.6 +/- 5.8<br>137.4 +/- 4.8	180,475<br>829,965	95.8%<br>86.5%	<0.001	0.161
1<br>2		0 - 0 mmol/L		180,480<br>830,011	95.8%<br>86.5%	<0.001	0.331

1<br>2	9028	Potassium [Moles/volume] in Serum, Plasma or Blood	4.0 +/- 0.7<br>4.1 +/- 0.6	177,456<br>817,172	94.2%<br>85.2%	<0.001	0.036
1<br>2		0 - 0 mmol/L		177,480<br>817,435	94.2%<br>85.2%	<0.001	0.299
1<br>2	9024	Creatinine [Mass/volume] in Serum, Plasma or Blood	1.9 +/- 2.6<br>1.5 +/- 3.9	179,737<br>800,211	95.4%<br>83.4%	<0.001	0.140
1<br>2		0 - 0 mg/dL		179,740<br>800,219	95.4%<br>83.4%	<0.001	0.397
1<br>2	9013	Hematocrit [Volume Fraction] of Blood	30.9 +/- 7.5<br>33.9 +/- 8.7	179,653<br>829,519	95.4%<br>86.5%	<0.001	0.372
1<br>2		0 - 0 %		179,655<br>829,578	95.4%<br>86.5%	<0.001	0.313
1<br>2	9018	Neutrophils [#/volume] in Blood	347.3 +/- 2242.0<br>141.8 +/- 1273.3	149,316<br>694,967	79.3%<br>72.5%	<0.001	0.113
1<br>2		0 - 0 10*3/uL		149,320<br>694,972	79.3%<br>72.5%	<0.001	0.160
1<br>2	9016	Lymphocytes/100 leukocytes in Blood	13.2 +/- 11.8<br>16.7 +/- 12.5	171,307<br>763,731	90.9%<br>79.6%	<0.001	0.286
1<br>2		0 - 0 %		171,308<br>763,734	90.9%<br>79.6%	<0.001	0.323
1<br>2	9017	Monocytes/100 leukocytes in Blood	7.2 +/- 4.6<br>7.9 +/- 4.2	174,347<br>778,787	92.6%<br>81.2%	<0.001	0.153
1<br>2		0 - 0 %		174,348<br>778,790	92.6%<br>81.2%	<0.001	0.341
1<br>2	9007	Eosinophils/100 leukocytes in Blood	1.6 +/- 2.6<br>1.8 +/- 2.6	171,603<br>755,785	91.1%<br>78.8%	<0.001	0.101
1<br>2		0 - 0 %		171,604<br>755,791	91.1%<br>78.8%	<0.001	0.349
1<br>2	9006	Basophils/100 leukocytes in Blood	0.4 +/- 0.5<br>0.4 +/- 0.6	170,871<br>747,640	90.7%<br>78.0%	<0.001	0.116
1<br>2		0 - 0 %		170,872<br>747,652	90.7%<br>78.0%	<0.001	0.357

Cohort 1 (N = 188,365) and cohort 2 (N = 188,365) characteristics after propensity score matching

Demographics

Cohort			Mean ± SD	Patients	% of Cohort	P-Value	Std diff.
1<br>2	Age	Current Age	67.8 +/- 15.7<br>67.6 +/- 16.7	188,365<br>188,365	100%<br>100%	<0.001	0.015
1<br>2	AI	Age at Index	63.6 +/- 16.1<br>63.3 +/- 17.1	188,365<br>188,365	100%<br>100%	<0.001	0.017
1<br>2	F	Female		81,722<br>80,666	43.4%<br>42.8%	0.001	0.011
1<br>2	2054-5	Black or African American		30,850<br>29,322	16.4%<br>15.6%	<0.001	0.022
1<br>2	M	Male		99,107<br>99,750	52.6%<br>53.0%	0.036	0.007
1<br>2	2106-3	White		113,392<br>113,736	60.2%<br>60.4%	0.252	0.004
1<br>2	1002-5	American Indian or Alaska Native		602<br>639	0.3%<br>0.3%	0.293	0.003

1<br>2	UNK	Unknown Race		18,471<br>18,388	9.8%<br>9.8%	0.649	0.001
1<br>2	2076-8	Native Hawaiian or Other Pacific Islander		2,052<br>1,980	1.1%<br>1.1%	0.254	0.004
1<br>2	UN	Unknown Ethnicity		30,758<br>29,730	16.3%<br>15.8%	<0.001	0.015
1<br>2	2186-5	Not Hispanic or Latino		139,530<br>141,019	74.1%<br>74.9%	<0.001	0.018
1<br>2	2135-2	Hispanic or Latino		18,077<br>17,616	9.6%<br>9.4%	0.010	0.008
1<br>2	2131-1	Other Race		7,685<br>7,653	4.1%<br>4.1%	0.792	0.001
1<br>2	2028-9	Asian		15,313<br>16,647	8.1%<br>8.8%	<0.001	0.025

Diagnosis

Cohort			Mean ± SD	Patients	% of Cohort	P-Value	Std diff.
1<br>2	E00-E89	Endocrine, nutritional and metabolic diseases		170,936<br>167,920	90.7%<br>89.1%	<0.001	0.053
1<br>2	K00-K95	Diseases of the digestive system		157,787<br>158,442	83.8%<br>84.1%	0.004	0.009
1<br>2	I00-I99	Diseases of the circulatory system		168,750<br>166,680	89.6%<br>88.5%	<0.001	0.035
1<br>2	N00-N99	Diseases of the genitourinary system		156,778<br>155,279	83.2%<br>82.4%	<0.001	0.021
1<br>2	J00-J99	Diseases of the respiratory system		151,860<br>150,787	80.6%<br>80.1%	<0.001	0.014
1<br>2	M00-M99	Diseases of the musculoskeletal system and connective tissue		130,595<br>128,866	69.3%<br>68.4%	<0.001	0.020
1<br>2	A00-B99	Certain infectious and parasitic diseases		169,408<br>167,730	89.9%<br>89.0%	<0.001	0.029
1<br>2	G00-G99	Diseases of the nervous system		135,290<br>134,285	71.8%<br>71.3%	<0.001	0.012
1<br>2	F01-F99	Mental, Behavioral and Neurodevelopmental disorders		115,170<br>114,291	61.1%<br>60.7%	0.003	0.010
1<br>2	C00-D49	Neoplasms		85,052<br>85,085	45.2%<br>45.2%	0.914	<0.001
1<br>2	V00-Y99	External causes of morbidity		78,730<br>78,703	41.8%<br>41.8%	0.929	<0.001
1<br>2	Q00-Q99	Congenital malformations, deformations and chromosomal abnormalities		22,924<br>22,849	12.2%<br>12.1%	0.708	0.001

Medication

Cohort			Mean ± SD	Patients	% of Cohort	P-Value	Std diff.
1<br>2	CN000	CENTRAL NERVOUS SYSTEM MEDICATIONS		178,513<br>175,837	94.8%<br>93.3%	<0.001	0.060
1<br>2	RE000	RESPIRATORY TRACT MEDICATIONS		177,141<br>174,945	94.0%<br>92.9%	<0.001	0.047

1<br>2	GA000	GASTROINTESTINAL MEDICATIONS		176,099<br>174,072	93.5%<br>92.4%	<0.001	0.042
1<br>2	AM000	ANTIMICROBIALS		173,956<br>171,382	92.4%<br>91.0%	<0.001	0.049
1<br>2	CV000	CARDIOVASCULAR MEDICATIONS		172,644<br>171,190	91.7%<br>90.9%	<0.001	0.027
1<br>2	HS000	HORMONES/SYNTHETICS/ MODIFIERS		170,538<br>168,895	90.5%<br>89.7%	<0.001	0.029
1<br>2	GU000	GENITOURINARY MEDICATIONS		157,804<br>157,741	83.8%<br>83.7%	0.781	0.001
1<br>2	MS000	MUSCULOSKELETAL MEDICATIONS		134,090<br>134,825	71.2%<br>71.6%	0.008	0.009
1<br>2	AP000	ANTIPARASITICS		10,564<br>10,477	5.6%<br>5.6%	0.537	0.002
1<br>2	AN000	ANTINEOPLASTICS		33,939<br>33,991	18.0%<br>18.0%	0.826	0.001

Laboratory

Cohort			Mean ± SD	Patients	% of Cohort	P-Value	Std diff.
1<br>2	9029	Sodium [Moles/volume] in Serum, Plasma or Blood	136.6 +/- 5.8<br>137.2 +/- 4.9	180,475<br>176,659	95.8%<br>93.8%	<0.001	0.116
1<br>2		0 - 0 mmol/L		180,480<br>176,665	95.8%<br>93.8%	<0.001	0.091
1<br>2	9028	Potassium [Moles/volume] in Serum, Plasma or Blood	4.0 +/- 0.7<br>4.1 +/- 0.6	177,456<br>173,636	94.2%<br>92.2%	<0.001	0.033
1<br>2		0 - 0 mmol/L		177,480<br>173,662	94.2%<br>92.2%	<0.001	0.081
1<br>2	9024	Creatinine [Mass/volume] in Serum, Plasma or Blood	1.9 +/- 2.6<br>1.6 +/- 3.2	179,737<br>176,713	95.4%<br>93.8%	<0.001	0.120
1<br>2		0 - 0 mg/dL		179,740<br>176,717	95.4%<br>93.8%	<0.001	0.071
1<br>2	9013	Hematocrit [Volume Fraction] of Blood	30.9 +/- 7.5<br>34.0 +/- 7.6	179,653<br>175,862	95.4%<br>93.4%	<0.001	0.415
1<br>2		0 - 0 %		179,655<br>175,866	95.4%<br>93.4%	<0.001	0.087
1<br>2	9018	Neutrophils [#/volume] in Blood	347.3 +/- 2242.0<br>276.3 +/- 1871.1	149,316<br>146,683	79.3%<br>77.9%	<0.001	0.034
1<br>2		0 - 0 10*3/uL		149,320<br>146,686	79.3%<br>77.9%	<0.001	0.034
1<br>2	9016	Lymphocytes/100 leukocytes in Blood	13.2 +/- 11.8<br>16.0 +/- 12.5	171,307<br>168,032	90.9%<br>89.2%	<0.001	0.227
1<br>2		0 - 0 %		171,308<br>168,033	90.9%<br>89.2%	<0.001	0.058
1<br>2	9017	Monocytes/100 leukocytes in Blood	7.2 +/- 4.6<br>7.8 +/- 4.3	174,347<br>171,225	92.6%<br>90.9%	<0.001	0.130
1<br>2		0 - 0 %		174,348<br>171,226	92.6%<br>90.9%	<0.001	0.060
1<br>2	9007	Eosinophils/100 leukocytes in Blood	1.6 +/- 2.6<br>1.8 +/- 2.7	171,603<br>169,130	91.1%<br>89.8%	<0.001	0.083
1<br>2		0 - 0 %		171,604<br>169,132	91.1%<br>89.8%	<0.001	0.045

1<br>2	9006	Basophils/100 leukocytes in Blood	0.4 +/- 0.5<br>0.4 +/- 0.5	170,871<br>168,351	90.7%<br>89.4%	<0.001	0.097
1<br>2		0 - 0 %		170,872<br>168,352	90.7%<br>89.4%	<0.001	0.045

Results

Results are summarized in the tables below. Outcomes analysis was performed on the cohorts after propensity score matching.

1 Mortality

Risk analysis

Cohort		Patients in cohort	Patients with outcome	Risk
1	main with albumin-age restriction	188,365	46,467	0.247
2	main without albumin-age restriction	188,365	27,814	0.148

		95% CI	z	p
**Risk Difference**	0.099	(0.097, 0.102)	76.383	0
**Risk Ratio**	1.671	(1.648, 1.693)	N/A	N/A
**Odds Ratio**	1.890	(1.859, 1.922)	N/A	N/A

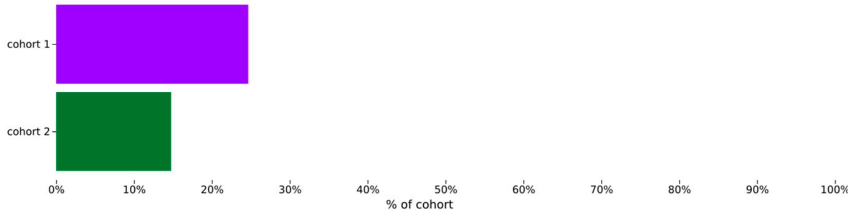

Kaplan - Meier survival analysis

Cohort		Patients in cohort	Patients with outcome	Median survival (days)	Survival probability at end of time window
1	main with albumin-age restriction	188,365	46,467	--	73.85%
2	main without albumin-age restriction	188,365	27,814	--	84.43%

	χ^2	df	p
**Log-Rank Test**	6221.143	1	0

	Hazard Ratio	95% CI	χ^2	df	p
**Hazard Ratio and Proportionality**	1.800	(1.774, 1.827)	77.560	1	0.000

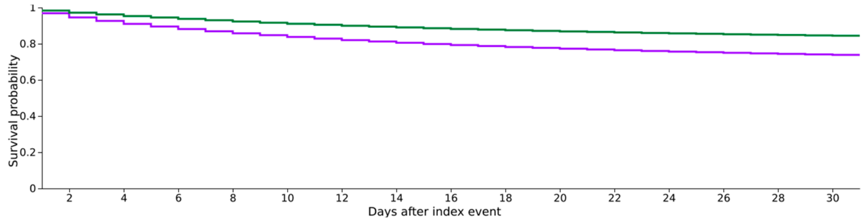

2 Shock

Risk analysis

Cohort		Patients in cohort	Patients with outcome	Risk
1	main with albumin-age restriction	188,365	23,669	0.126
2	main without albumin-age restriction	188,365	17,235	0.091

		95% CI	z	p
**Risk Difference**	0.034	(0.032, 0.036)	33.694	0.000
**Risk Ratio**	1.373	(1.348, 1.399)	N/A	N/A
**Odds Ratio**	1.427	(1.398, 1.457)	N/A	N/A

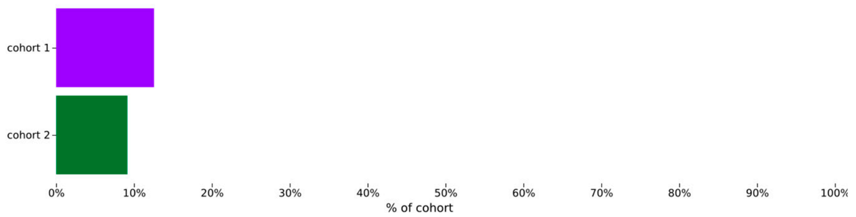

Kaplan - Meier survival analysis

Cohort		Patients in cohort	Patients with outcome	Median survival (days)	Survival probability at end of time window
1	main with albumin-age restriction	188,365	23,669	--	86.21%
2	main without albumin-age restriction	188,365	17,235	--	90.30%

	χ^2	df	p
**Log-Rank Test**	1335.977	1	0.000

	Hazard Ratio	95% CI	χ²	df	p
**Hazard Ratio and Proportionality**	1.436	(1.409, 1.465)	69.381	1	0.000

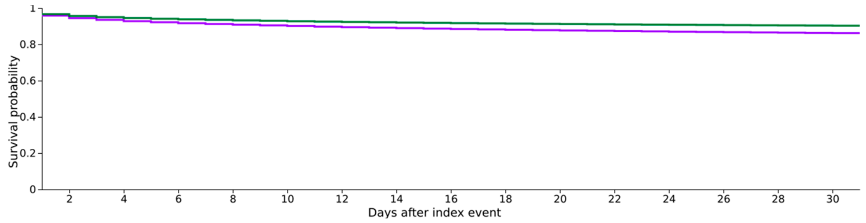

3 Septic shock

Risk analysis

Cohort		Patients in cohort	Patients with outcome	Risk
1	main with albumin-age restriction	188,365	18,964	0.101
2	main without albumin-age restriction	188,365	14,257	0.076

		95% CI	z	p
**Risk Difference**	0.025	(0.023, 0.027)	27.045	0.000
**Risk Ratio**	1.330	(1.303, 1.358)	N/A	N/A
**Odds Ratio**	1.367	(1.336, 1.399)	N/A	N/A

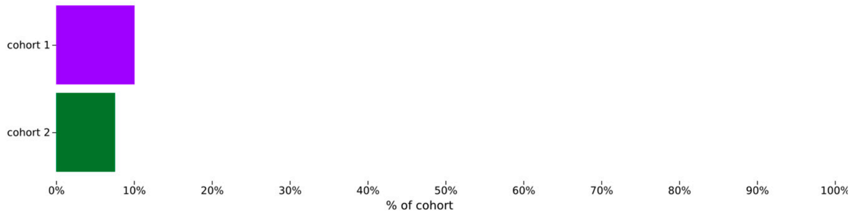

Kaplan - Meier survival analysis

Cohort		Patients in cohort	Patients with outcome	Median survival (days)	Survival probability at end of time window
1	main with albumin-age restriction	188,365	18,964	--	88.93%
2	main without albumin-age restriction	188,365	14,257	--	91.98%

	χ²	df	p
**Log-Rank Test**	875.540	1	0.000

	Hazard Ratio	95% CI	χ²	df	p

	Hazard Ratio	95% CI	χ²	df	p
**Hazard Ratio and Proportionality**	1.384	(1.355, 1.415)	72.590	1	0.000

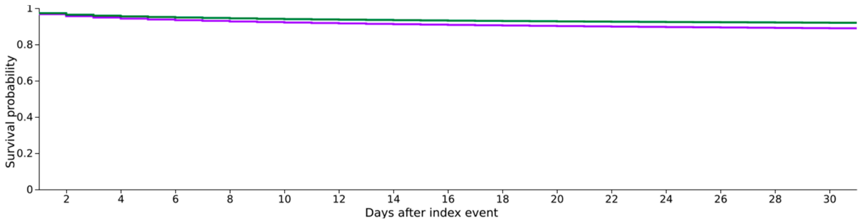

4 Hypovolemic shock

Risk analysis

Cohort		Patients in cohort	Patients with outcome	Risk
1	main with albumin-age restriction	188,365	1,785	0.009
2	main without albumin-age restriction	188,365	1,242	0.007

		95% CI	z	p
**Risk Difference**	0.003	(0.002, 0.003)	9.909	0.000
**Risk Ratio**	1.437	(1.337, 1.545)	N/A	N/A
**Odds Ratio**	1.441	(1.340, 1.550)	N/A	N/A

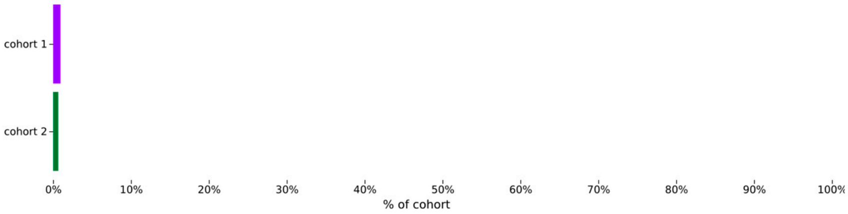

Kaplan - Meier survival analysis

Cohort		Patients in cohort	Patients with outcome	Median survival (days)	Survival probability at end of time window
1	main with albumin-age restriction	188,365	1,785	--	98.93%
2	main without albumin-age restriction	188,365	1,242	--	99.29%

	χ²	df	p
**Log-Rank Test**	120.435	1	0.000

	Hazard Ratio	95% CI	χ²	df	p

**Hazard Ratio and Proportionality**	1.496	(1.391, 1.608)	3.804	1	0.051

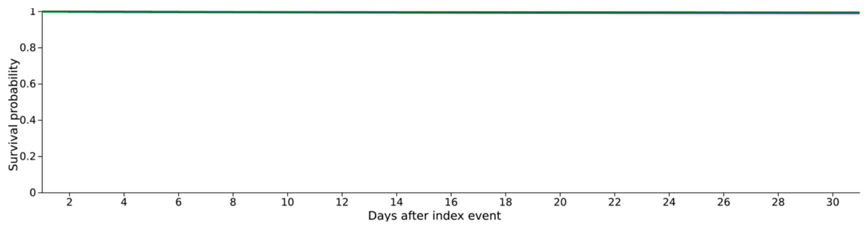

5 Cardiogenic shock

Risk analysis

Cohort		Patients in cohort	Patients with outcome	Risk
1	main with albumin-age restriction	188,365	3,450	0.018
2	main without albumin-age restriction	188,365	2,299	0.012

		95% CI	z	p
**Risk Difference**	0.006	(0.005, 0.007)	15.297	0.000
**Risk Ratio**	1.501	(1.424, 1.581)	N/A	N/A
**Odds Ratio**	1.510	(1.432, 1.592)	N/A	N/A

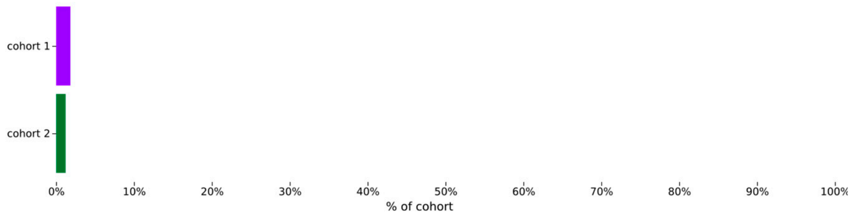

Kaplan - Meier survival analysis

Cohort		Patients in cohort	Patients with outcome	Median survival (days)	Survival probability at end of time window
1	main with albumin-age restriction	188,365	3,450	--	97.98%
2	main without albumin-age restriction	188,365	2,299	--	98.69%

	χ^2	df	p
**Log-Rank Test**	272.091	1	0.000

	Hazard Ratio	95% CI	χ^2	df	p

**Hazard Ratio and Proportionality**	1.553	(1.473, 1.637)	0.000	1	0.996

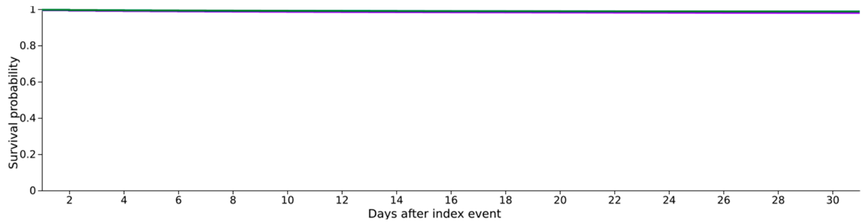

6 Heart failure

Risk analysis

Cohort		Patients in cohort	Patients with outcome	Risk
1	main with albumin-age restriction	188,365	28,969	0.154
2	main without albumin-age restriction	188,365	27,484	0.146

		95% CI	z	p
**Risk Difference**	0.008	(0.006, 0.010)	6.779	0.000
**Risk Ratio**	1.054	(1.038, 1.070)	N/A	N/A
**Odds Ratio**	1.064	(1.045, 1.083)	N/A	N/A

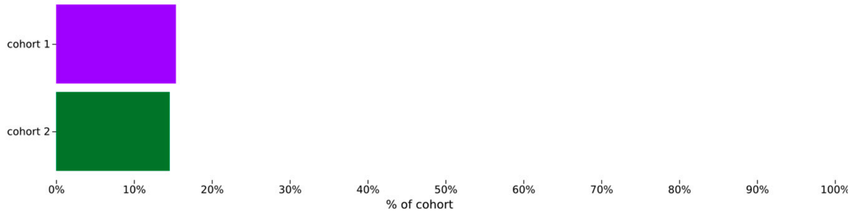

Kaplan - Meier survival analysis

Cohort		Patients in cohort	Patients with outcome	Median survival (days)	Survival probability at end of time window
1	main with albumin-age restriction	188,365	28,969	--	82.49%
2	main without albumin-age restriction	188,365	27,484	--	84.21%

	χ^2	df	p
**Log-Rank Test**	125.222	1	0.000

	Hazard Ratio	95% CI	χ^2	df	p

**Hazard Ratio and Proportionality**	1.098	(1.080, 1.116)	168.034	1	0.000

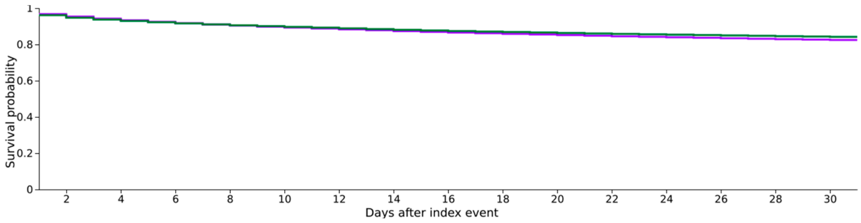

7 Pulmonary edema

Risk analysis

Cohort		Patients in cohort	Patients with outcome	Risk
1	main with albumin-age restriction	188,365	12,188	0.065
2	main without albumin-age restriction	188,365	8,658	0.046

		95% CI	z	p
**Risk Difference**	0.019	(0.017, 0.020)	25.155	0.000
**Risk Ratio**	1.408	(1.370, 1.446)	N/A	N/A
**Odds Ratio**	1.436	(1.396, 1.477)	N/A	N/A

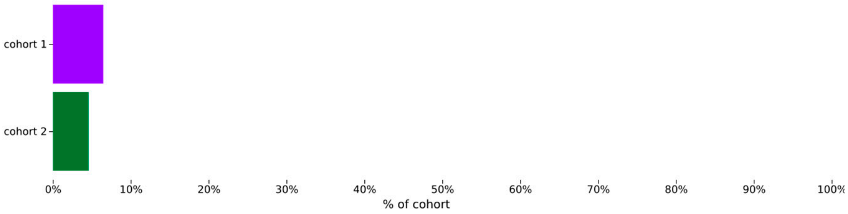

Kaplan - Meier survival analysis

Cohort		Patients in cohort	Patients with outcome	Median survival (days)	Survival probability at end of time window
1	main with albumin-age restriction	188,365	12,188	--	92.69%
2	main without albumin-age restriction	188,365	8,658	--	95.02%

	χ^2	df	p
**Log-Rank Test**	785.826	1	0.000

	Hazard Ratio	95% CI	χ^2	df	p

**Hazard Ratio and Proportionality**	1.479	(1.438, 1.520)	4.924	1	0.026

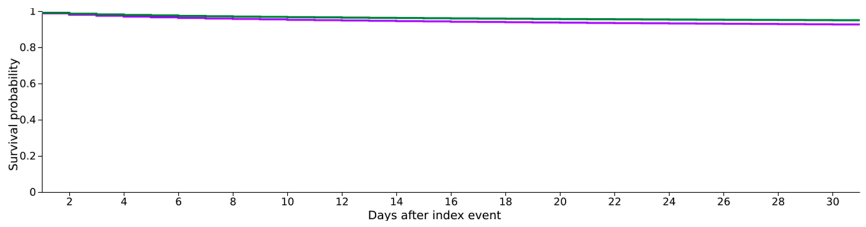

Appendix A – Text Representation of the Cohorts Definition

This section lists all terms used in the definitions of the two cohorts.

Query Criteria for Cohort 1 (query name: main with albumin-age restriction)

Patients must have:
  Age (Age) (at least 18 years (most recent occurrence)).

All the following must be satisfied:

  Sepsis with hypoalbuminemia: The terms in this group occurred on or after Apr 1, 2014
    Patients must have:
      all of the following:
        Sepsis, unspecified organism (UMLS:ICD10CM:A41.9); and
        Albumin [Mass/volume] in Serum, Plasma or Blood (TNX:9045) (between 0.00 and 3.50 g/dL).
    Patients cannot have:
      Shock, unspecified (UMLS:ICD10CM:R57.9).
  albumin infusion: Any instance of albumin infusion occurred on or after any instance of Sepsis with hypoalbuminemia
    Patients must have:
      any of the following:
        albumin,aggregated (NLM:RXNORM:317176); or
        albumin human, USP (NLM:RXNORM:828529); or
        albumin microspheres, human (NLM:RXNORM:1370439); or
        serum albumin, bovine (NLM:RXNORM:1426381); or
        albumin,iodinated I-131 serum (NLM:RXNORM:314301); or
        albumin tannate (NLM:RXNORM:OMOP4819585).

Query Criteria for Cohort 2 (query name: main without albumin-age restriction)

Patients must have:
  Age (Age) (at least 18 years (most recent occurrence)).

All the following must be satisfied:

  Sepsis with hypoalbuminemia: The terms in this group occurred on or after Apr 1, 2014
    Patients must have:
      all of the following:
        Sepsis, unspecified organism (UMLS:ICD10CM:A41.9); and
        Albumin [Mass/volume] in Serum, Plasma or Blood (TNX:9045) (between 0.00 and 3.50 g/dL).
    Patients cannot have:
      Shock, unspecified (UMLS:ICD10CM:R57.9).
  albumin infusion: Any instance of albumin infusion occurred on or after any instance of Sepsis with hypoalbuminemia
    Patients cannot have:
      all of the following:
        albumin,aggregated (NLM:RXNORM:317176); and
        albumin microspheres, human (NLM:RXNORM:1370439); and
        albumin tannate (NLM:RXNORM:OMOP4819585); and

albumin,iodinated I-131 serum (NLM:RXNORM:314301); and
albumin human, USP (NLM:RXNORM:828529); and
serum albumin, bovine (NLM:RXNORM:1426381).

Appendix B – Text Representation of the Analysis Setup

This section contains the Index Event definition for each cohort.

The index event for Cohort 1 (query name: main with albumin-age restriction) is defined as the following:

All the following must be satisfied:

Sepsis with hypoalbuminemia: The terms in this group occurred on or after Apr 1, 2014
Patients must have:
all of the following:
Sepsis, unspecified organism (UMLS:ICD10CM:A41.9); and
Albumin [Mass/volume] in Serum, Plasma or Blood (TNX:9045) (between 0.00 and 3.50 g/dL).
Patients cannot have:
Shock, unspecified (UMLS:ICD10CM:R57.9).
albumin infusion: Any instance of albumin infusion occurred on or after any instance of Sepsis with hypoalbuminemia
Patients must have:
any of the following:
albumin,aggregated (NLM:RXNORM:317176); or
albumin human, USP (NLM:RXNORM:828529); or
albumin microspheres, human (NLM:RXNORM:1370439); or
serum albumin, bovine (NLM:RXNORM:1426381); or
albumin,iodinated I-131 serum (NLM:RXNORM:314301); or
albumin tannate (NLM:RXNORM:OMOP4819585).

The index event for Cohort 2 (query name: main without albumin-age restriction) is defined as the following:

All the following must be satisfied:

Sepsis with hypoalbuminemia: The terms in this group occurred on or after Apr 1, 2014
Patients must have:
all of the following:
Sepsis, unspecified organism (UMLS:ICD10CM:A41.9); and
Albumin [Mass/volume] in Serum, Plasma or Blood (TNX:9045) (between 0.00 and 3.50 g/dL).
Patients cannot have:
Shock, unspecified (UMLS:ICD10CM:R57.9).
albumin infusion: Any instance of albumin infusion occurred on or after any instance of Sepsis with hypoalbuminemia
Patients cannot have:
all of the following:
albumin,aggregated (NLM:RXNORM:317176); and
albumin microspheres, human (NLM:RXNORM:1370439); and
albumin tannate (NLM:RXNORM:OMOP4819585); and
albumin,iodinated I-131 serum (NLM:RXNORM:314301); and

albumin human, USP (NLM:RXNORM:828529); and
serum albumin, bovine (NLM:RXNORM:1426381).

Appendix C – Text Representation of the Outcomes Definition

This analysis includes the following outcomes:

Mortality
- Patients must have:
  - any of the following:
    - Deceased (Deceased); or
    - Ill-defined and unknown cause of mortality (UMLS:ICD10CM:R99).

Shock
- Patients must have:
  - any of the following:
    - Shock, unspecified (UMLS:ICD10CM:R57.9); or
    - Shock, not elsewhere classified (UMLS:ICD10CM:R57); or
    - Severe sepsis with septic shock (UMLS:ICD10CM:R65.21); or
    - Hypovolemic shock (UMLS:ICD10CM:R57.1); or
    - Cardiogenic shock (UMLS:ICD10CM:R57.0).

Septic shock
- Patients must have:
  - Severe sepsis with septic shock (UMLS:ICD10CM:R65.21).

Hypovolemic shock
- Patients must have:
  - Hypovolemic shock (UMLS:ICD10CM:R57.1).

Cardiogenic shock
- Patients must have:
  - Cardiogenic shock (UMLS:ICD10CM:R57.0).

Heart failure
- Patients must have:
  - any of the following:
    - Heart failure (UMLS:ICD10CM:I50); or
    - Left ventricular failure, unspecified (UMLS:ICD10CM:I50.1); or
    - Heart failure, unspecified (UMLS:ICD10CM:I50.9); or
    - Unspecified diastolic (congestive) heart failure (UMLS:ICD10CM:I50.30); or
    - Unspecified systolic (congestive) heart failure (UMLS:ICD10CM:I50.20).

Pulmonary edema
- Patients must have:
  - any of the following:
    - Pulmonary edema (UMLS:ICD10CM:J81); or
    - Acute pulmonary edema (UMLS:ICD10CM:J81.0).
